# Promising applications of electromagnetic field therapy in dental implantology: A systematic review

**DOI:** 10.34172/japid.2024.001

**Published:** 2024-01-06

**Authors:** Saba Khazeni, Xaniar Mohammadi Khanghah, Meghdad Eslami, Mohamadamin Ansari, Mohammad Hossein Asadi

**Affiliations:** Department of Maxillofacial Surgery, Faculty of Dentistry, Tabriz University of Medical Science, Tabriz, Iran

**Keywords:** Dental implants, Electromagnetic fields, Magnetic fields, Systematic review

## Abstract

**Background.:**

Non-ionizing electromagnetic field (EMF) exposure therapies are non-invasive and safe treatment options that can potentially change available treatments. In this review, we examined the applications of such therapies in dental implant surgery by conducting a systematic review.

**Methods.:**

A comprehensive search of several international electronic databases was conducted from inception to December 14, 2022. This review included interventional studies that evaluated the advantages of adjunctive magnetic or combined EMFs on dental implants compared to conventional treatments.

**Results.:**

From a total of 1695 studies, 12 preclinical and clinical studies were selected, discussing EMF-based treatments for enhancing implant stability, osteogenesis, and osseointegration, as well as alleviating post-implant surgery manifestations. Almost all studies on maxillary and mandibular implant stability showed beneficial effects of non-ionizing EMF in humans. Most studies evaluating osteogenesis and osseointegration indicated that EMF exposure could accelerate bone repair and peri-implant bone formation and increase bone contact ratios, bone volume fraction (bone volume/total volume), trabecular number, and trabecular thickness. Only two clinical studies examined the effect of EMF on pain and swelling after dental implant surgery, with one finding that subjects exposed to EMF used analgesics fewer times and in far lower doses than the control group and the other finding no significant difference in reducing these outcomes between the groups.

**Conclusion.:**

Overall, devices that deliver non-ionizing low-level EMF can be a viable and widely recognized non-invasive adjuvant therapy for attaining success and better outcomes after dental implant surgery due to their efficacy, safety, and short exposure time.

## Introduction

 Electromagnetic fields (EMF) are a combination of electric and magnetic fields created when electric charges move through an electric field.^1^ Exposure to EMFs can be beneficial or harmful, depending on the technical parameters that stimulate the target, such as frequency and field strength, as well as the characteristics of biological targets. Non-ionizing EMFs are low-level radiations that are mainly considered safe for human health. Despite some debate, those with frequencies in the static and extremely low frequency (ELF) ranges have revealed some health benefits.^2^

 In recent years, EMF-based approaches have been a topic of interest for managing some health problems.^3-5^ This is also true for dental conditions, where an experimental investigation by Matsumoto et al^6^ investigated the effects of EMF on bone formation surrounding dental implants for the first time in 2000. Subsequently, a number of preclinical and clinical studies have shown that EMF-based interventions can improve the success of dental implant surgery.^6-17^

 Numerous clinical and preclinical studies have evaluated the applications of EMF-based interventions on implant stability, peri-implant osteogenesis and osseointegration, and postoperative symptoms. However, upon observing the absence of a review of the evidence for these types of studies assessing EMF-based interventions in patients undergoing dental implants, we performed a systematic review to evaluate the efficacy of such interventions on outcomes above in experimental studies and clinical trials.

## Methods

 The Preferred Reporting Items for Systematic Reviews and Meta-Analyses (PRISMA) 2020 guideline was implemented when we reported the current study.^18^

## Study selection

 The research question was formulated as follows, per PICOST: 1) The population included patients undergoing dental implant surgery or preclinical models of the dental implant; (2) The intervention group underwent either magnetic or combined EMFs for therapeutic purposes; (3) The comparison group underwent either an inactive control or no treatment; (4) The outcome was an improvement in at least one measure of clinical or paraclinical endpoint related to dental implant surgery compared to the control group; (5) Only interventional studies, including clinical and preclinical research, were taken into account; (6) The search time was from the inception to December 2022.

 Two authors independently reviewed and selected the included studies. A third researcher was consulted when required. The following inclusion criteria were in place: (1) Interventional studies (clinical trials, in vitro, in vivo, and in-silico preclinical studies) published in English up to December 14, 2022, and (2) Studies evaluating the advantages of magnetic or combined EMFs on dental implants. Our review excluded non-English-language studies, observational studies, review articles, case reports, case series, conference abstracts, errata, and comments.

## Data sources and search strategy

 A comprehensive search of several international electronic databases was conducted from inception to December 14, 2022. PubMed, Scopus, and Embase were among the databases that were searched using the keywords “electromagnetic field,” “magnetic field therapy,” “magnetic field,” and “PEMF” combined with terms related to dental implant surgery, including “dental implant,” “osseointegration,” “peri-implant osteogenesis,” “bone-implant contact,” “dental prosthesis, implant-supported,” and “dental implant stability.” Before finalizing the retrieved papers, the search was undertaken once again to uncover any other identified studies. Furthermore, we examined the references of related papers to discover possibly missing research.

## Data extraction and risk of bias evaluation

 Two researchers extracted the necessary information using two separate sample extraction forms designed for different types of studies. Another author verified the accuracy of the extracted data. The following data were extracted from the selected papers: Authors’ names, publication year, study design, study population and experimental model, EMF characteristics, EMF exposure duration, site of EMF application, comparison group, outcomes, and main findings. Two researchers separately appraised the risk of bias in the included papers through the SYRCLE instrument^19^ and Cochrane Rob 2.0^20^ for preclinical and clinical studies, respectively.

## Study selection process

**Figure 1:**
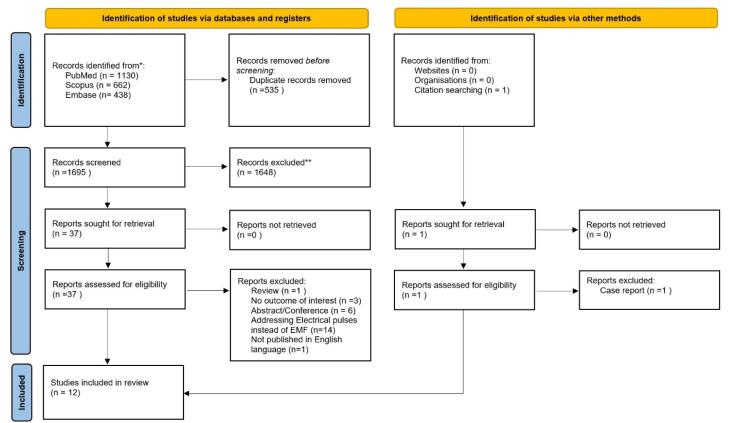
Prisma chart of the manuscript selection process

Figure 1 shows how the PRISMA flowchart was used to select eligible papers. Using the described methodology, 2230 references were acquired from our search. After examining the titles and abstracts, 535 duplicate publications and 1648 references were eliminated. The full texts of 37 references were examined. Following a thorough evaluation, 25 studies were excluded for various reasons. Twelve interventional studies^6-17^ were finally included in this review.

## Study characteristics

 The included studies were divided into three groups based on the outcomes related to dental implant surgery: (1)Implant stability, (2) ossification and osseointegration, and (3) postoperative presentations like pain and swelling. Four included studies examined the effect of EMFs on implant stability, all of which were clinical.^7,13,16,17^ Six preclinical research examined the impact of EMF-based therapies on either ossification or osseointegration, or both, five of which were in vitro,^6,9,11,14,15^ and two of which had an in vivo design.^8,14^ Finally, two studies investigated the effects of EMFs on postoperative presentations like pain and swelling, both of which were clinical.^10,12^

**Figure 2:**
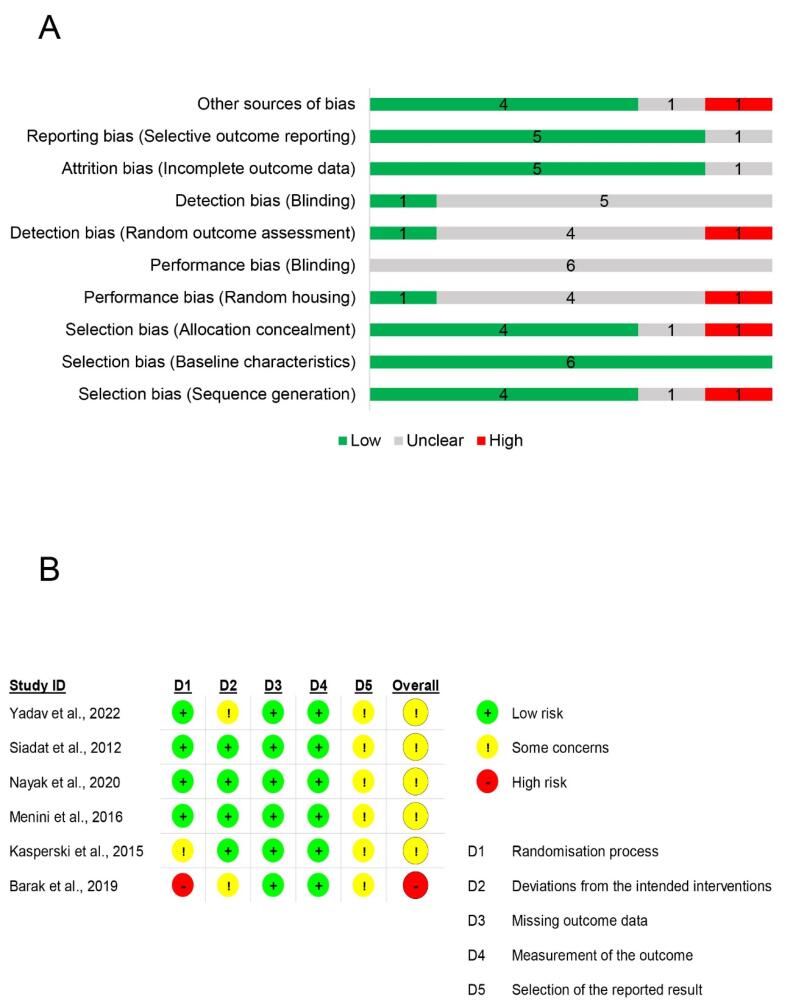
Risk of bias assessment. A: Risk of bias assessment in preclinical studies using SYRCLE’s RoB tool. B: Risk of bias assessment in clinical studies using Cochrane Rob 2.0

Figure 2A shows that detection and performance bias may be the most prevalent bias in the animal studies included. All the animal studies addressed selection bias adequately (baseline characteristics). In addition, the majority of the included preclinical studies (5 studies) addressed attrition and reporting bias. Only four studies addressed selection bias (sequence generation and allocation concealment). Figure 2B depicts the outcomes of the quality assessment of clinical studies. There was no clinical study with a low risk of bias; five had some concerns, and one had a high risk of bias. Domains 3 and 4 posed a low risk of bias in all studies. Regarding the randomization process in the first domain, one study raised some concerns, and another lacked randomization. Concerns exist in domain 5 for all trials, primarily due to the absence of a prespecified analysis plan.

## Main findings

 This section reviews each set of studies concerning the outcomes related to dental implant surgery and provides an overview of the major conclusions from each study.

###  Implants Stability

 In this section, we examine all the research that examined how EMFs affected the stability of implants in patients having undergone dental implant surgery. Table 1 provides characteristics of studies examining the impact of EMF on the stability of implants.

**Table 1:** Characteristics of included studies

**Author, year**	**Study design (N)**	**Intervention characteristics: Frequency/Field strength/pulse width**	**EMF exposure duration**	**Comparison group**	**Outcomes & main findings**
Barak et al^7^	Clinical study (28)	NR/NR/NR	24 hours a day for 50 days	Regular control healing caps	-Maxillary implants stability was significantly higher in the EMF group than controls at 15 and 50 days post-implantation -Mandibular implant stability was significantly higher in the EMF group than in controls at 30 days post-implantation
do Nascimento et al^8^	In vivo study in dogs (8)	1.5 MHz/0.8 mT/25 µs	20 minutes every day for 14 days	No treatment	EMF did not modify the bone repair surrounding dental implants as compared to the animals received no treatment
Grana et al^9^	In vitro study in rats (60)	50 Hz/72 mT/NR	Twice daily for 30-minute sessions	Sham sessions in an unplugged coil	The PEMF group had a greater area of ossification index 10 and 20 days after treatment than the control group.
Kasperski et al^10^	Clinical study (30)	0.1–100 Hz/NR/NR	Exposure to magnetic field for about 15 minutes before treatment and during the follow-up appointment 6 hours after implantation.	- Placebo - Magneto stimulation with LED therapy	- Patients exposed to EMF took analgesics fewer times and in far lower doses than the control group - “EMF” was superior to “magneto stimulation + LED” therapy in reducing pain.
Kim et al^11^	In vitro study on implant specimens in adult male New Zealand white rabbits (56)	0 Hz (Static)/15 mT/NR	Continuously for 8 weeks	No treatment with SMF	- SMFs enhanced bone volume fraction and trabecular number and thickness at 4 and 8 weeks of treatment, according to µCT information - Histological examination revealed that SMFs stimulated new bone growth and direct bony contact with implants at 4 and 8 weeks after treatment. - Microarray analysis identified the upregulation of genes, including extracellular matrix (ECM)-related genes (COL10A1, COL9A1, and COL12A1) and growth factor (GF)-related genes (CTGF and PDGFD) in response to SMFs
Matsumoto et al^6^	In vitro study on implant specimens in Japanese white rabbits (45)	100 Hz/ 0.2, mT, 0.3 mT or 0.8 mT/ 25 µs	Intermittently 4 h or 8 h daily for 1, 2 or 4 weeks after implantation	No treatment with PEMF	- The PEMF-treated samples had considerably higher bone contact ratios than the control samples. - The bone contact ratio and bone surface of 0.2 mT- and 0.3 mT-stimulated groups were considerably greater than their respective values for the group stimulated with 0.8 mT. - There was no considerable variation in bone contact ratio or bone region between 4 hours and 8 hours per day of PEMF application. - The 2-week treated group had much more bone developed surrounding the implant than the 1-week treated group, but there was no considerable variation between the 2-week and 4-week stimulated groups.
Menini et al^12^	double-blind, randomized placebo-controlled clinical study (11)	1 kHz /100 mV/cm/ NR	Intermittently for 48 h after surgery	Sham (inactive field)	- No statistically considerable variation was observed between the active and control sides for swelling or pain
Nayak et al^13^	Randomized controlled clinical study (19 subjects, 40 implants in total)	10–50 kHz/ 0.05–0.5 mT/NR	Continuously for 30 days	Sham healing cap	- The PEMF group (miniaturized electromagnetic device) presented higher implant stability quotient mean values as compared to the sham group after 12 weeks - Primary stability and overall stability increased in the MED ( + 6.8% and + 13.0%) but decreased in the control group (-7.6% and -2.0%) compared to baseline values - During the first four weeks, TNF-alpha levels were lower in the group treated with PEMF than in the sham group
Nunes et al^14^	In vivo and in vitro study of male Wistar rats (60)	15 Hz/ ± 1 mT/200 μs	1 or 3 hours per day, 5 days/week until euthanasia (max 45 days)	The control animals were caged to imitate stimulation and stress, but the PEMF device was turned off.	-PEMF for 1 hour per day showed better results compared with PEMF for 3 hours per day in bone volume and density, cell survival, total protein content, and mineralization nodules - PEMF for 3 hours per day demonstrated superior results in trabecular bone thickness and cell proliferation compared with PEMF for 1 hour per day, especially at osseointegration early periods - There were no differences in histomorphometry analysis between the PEMF groups. - BIC and the number of trabeculae were higher in PEMF-treated groups compared to the control group, but there were no significant differences in these parameters between the two PEMF-treated groups.
Özen et al^15^	In vitro study in the rabbit mandibular model (28)	100 Hz/ 0.2 mT/ 25 µs	4 hours per day for 2 consecutive weeks	No treatment with PEMF	- There was no significant difference in bone osteoblastic activity, new trabecular bone, or fibrous tissue production between the control and PEMF-treated groups. - At week 8, there were considerable variations in bone osteoblastic activity and new trabecular bone growth between the control and PEMF-stimulated groups.
Siadat et al^16^	Randomized controlled clinical study (20)	0 HZ (static)/NR/NT	Continuously for 3 months	Conventional healing abutment	-Radiofrequency analysis showed significantly greater stability for implants in the active group than that of the sham group following 1 month - At month 2, less crestal bone loss was found in the test group -There was no discernible difference between the groups for each measure at month 3.
Yadav et al^17^	Double-blinded randomized controlled clinical study (40)	0 HZ (static)/ NR/ NR	Continuously for 4 months	Conventional healing cap	- At 2, 3, and 4 months, osseointegration and implant stability were considerably higher in subjects with magnetic healing cap insertion than in groups with conventional healing caps.

BIC, Bone implant contact; EMF, electromagnetic field; PEMF, pulsed electromagnetic field; N, number; NR, not reported; MED, miniaturized electromagnetic device.

 Barak et al^7^ evaluated the efficacy of the miniaturized electromagnetic device (MED) on the stability of the implant for the first time in 12 human subjects divided into two study groups: 1) implants with MED healing caps (n = 12) and 2) implants with regular control healing caps (n = 16). Resonance frequency analysis (RFA) was applied to assess implant stability. The researchers discovered the following benefits of MED: 1) 15, 30, and 50 days after implantation, MED healing caps dramatically increased the stability of maxillary implants compared to controls. 2) 30 days after implantation, the stability of mandibular implants was much higher with MED healing caps than with control caps.

 In a randomized clinical trial, Nayak et al^13^ examined the effects of pulsed electromagnetic field (PEMF) on implant stability using RFA. In the current investigation, 19 participants with 40 implants were randomly assigned to either the PEMF group, which received MED, or the control group, which received a sham healing cap. The PEMF group showed greater mean values for the implant stability quotient than the control group within the first two weeks. Additionally, in the first four weeks, the group receiving MED therapy had lower TNF- levels than the control group.

 Siadat et al^16^ designed a randomized clinical study to assess the impact of static magnetic fields (SMFs) immediately placed in fresh extraction sockets on the stability of dental implants. After one month, RFA assessments revealed considerably higher implant stability in the active group than in the control group.

 Yadav et al^17^ designed a double-blinded, randomized, controlled study to determine if SMF generated with safer magnets promoted implant stability and osseointegration. Forty subjects were divided into two groups: (1) magnetic healing caps (n = 20) and (2) conventional healing caps (n = 20). Compared to implants with a traditional healing cap, they demonstrated noticeably increased implant stability and osseointegration in implants activated by SMF while utilizing a magnetic healing cap.

 Overall, in clinical studies, SMF, ELF, and intermediate frequency (IF) EMF improved implant stability. More randomized clinical trials with larger sample sizes are needed, as is the control of potentially confounding variables in exposure protocols.

###  Ossification and osseointegration

 This section summarizes all the studies examining the impact of EMFs on either ossification, osseointegration, or both. Table 1 lists the characteristics of the studies.

 Do Nascimento et al^8^ assessed the efficacy of a continuous EMF on bone regeneration surrounding dental implants in dogs. They discovered newly produced bone in the space between the implant surface and alveolar bone in both groups, albeit in modest amounts, but no considerable difference was found between the two groups.

 Grana et al^9^ investigated whether short-term exposure to PEMF stimulates bone repair and peri-implant bone growth in rats. They found that the ossification index was higher in the animals in the PEMF group than in the sham group 10 days following placement in the right tibial crest.

 Kim et al^11^ used µCT, histologic analyses, microarrays, and quantitative real-time polymerase chain reaction (qRT-PCR ) to assess the effects of SMFs on bone regeneration surrounding titanium implants. SMFs enhanced bone volume fraction, defined as the ratio of bone volume to total volume, trabecular number, and trabecular thickness, according to µCT data. The histology findings revealed that SMFs encouraged new bone growth and direct bone-to-implant contact. According to a microarray study, 293 genes were elevated ( > 2-fold) in response to SMFs. The overexpression of extracellular matrix-related genes and growth factor-related genes was validated by quantitative real-time PCR (qRT-PCR). The mitogen-activated protein kinase (MAPK), Wnt, and PPAR-gamma signaling pathways were found to be involved in implant healing by Gene Ontology and pathway analysis.

 Matsumoto et al^6^ investigated the effect of PEMF on bone growth surrounding a dental implant with a rough surface in rabbits. They discovered that the PEMF-stimulated animals had considerably greater bone contact ratios than the control group. The bone surface ratio and bone region of the groups treated with 0.2 and 0.3 mT were noticeably higher than the corresponding values for the groups treated with 0.8 mT. There was no statistically considerable variation in bone surface ratio or bone region between 4 and 8 hours of PEMF therapy daily. The two-week treated animals had much more bone surrounding the implant than the 1-week treated groups, but there was no discernible difference between the two-week and four-week treated groups.

 Nunes et al^14^ examined the efficacy of two PEMF protocols on osseointegration to determine which parameters are best for its use in dentistry, particularly in optimizing the implant osseointegration process. Compared to three hours of PEMF per day, one hour of PEMF per day yielded better outcomes in bone volume and density, cell survival, total protein content, and mineralization nodules. PEMF for three hours per day performed better in terms of trabecular bone density and cell proliferation than PEMF for one hour per day, especially during the early stages of osseointegration. The histomorphometry analyses of the PEMF groups were identical. There were more trabeculae and bone‒implant contact (BIC) in the PEMF-treated groups than in the control group. However, these parameters did not differ significantly between the two PEMF-treated groups.

 Özen et al^15^ studied the impact of PEMFs on bone development following titanium dental implant insertion in a rabbit mandibular model. There was no statistically significant change in osteoblastic activity, new trabecular bone, or fibrous tissue production between the control and PEMF-treated groups. At week 8, however, there were substantial changes in bone osteoblastic activity and new trabecular bone growth between the control and PEMF-treated groups.

 In summary, six preclinical studies examined the effects of EMF on ossification and osseointegration, most of which found beneficial effects.

###  Postoperative presentations like pain and swelling

 Two clinical trials, summarized in Table 1 and discussed below, examined the impact of EMF on post-implant surgical symptoms such as pain and swelling.

 Kasperski et al^10^ investigated the analgesic efficacy of magneto stimulation and magneto-led therapy following implant treatment. EMF patients used analgesics less frequently and with substantially weaker analgesics than the control group. Compared to magneto stimulation with LED therapy, EMF produced better results in terms of pain relief.

 Menini et al^12^ investigated if PEMF can enhance postoperative swelling and pain control following the immediate loading of a complete arch implant. No statistically significant differences in swelling or pain were detected between the test and control sides.

## Discussion

 This review summarized preclinical and clinical studies investigating the benefits of EMF-based interventions on outcomes related to dental implant surgery, including implant stability, ossification and osseointegration, and postoperative presentations like pain and swelling. Most studies showed that non-ionizing EMFs could be a safe and novel therapeutic approach for improving outcomes related to dental implant surgery, specifically as adjuncts to common treatments.

 EMF-based therapies are gaining popularity due to their many advantages over conventional treatments. Contrary to surgical treatments, these approaches are non-invasive and can be administered quickly, which might increase patients’ adherence to this treatment. Moreover, the safety of EMF exposure has been well-established in many studies.^1^ All these findings prompted researchers to broaden their understanding of the potential advantages and mechanisms of action of this promising new therapeutic approach.

 All the studies included in this review used low-level EMF, which has been known as a safe and effective option for various diseases.^4,21^ This is also true for dental implantology, where multiple preclinical and clinical studies have indicated the beneficial effects of EMF-based interventions, primarily in the static and ELF range, for some outcomes, including implant stability, ossification and osseointegration, and postoperative presentations like pain and swelling. The mechanism of therapeutic effects varied based on the frequency, field strength, site of exposure, and other EMF features.

 We also examined the quality of the included articles and found some limitations and information gaps. Some studies had low scores based on our quality assessment, leading to inappropriate and imprecise results. One clinical study had no control group and only compared the results between two active groups, which can produce a bias. Most studies did not evaluate the safety of the intervention in the short and long term. Also, the long-term efficacy of EMF exposure was not investigated. Almost all studies used completely different EMFs, which varied in frequency, field strength, treatment regimen duration, site of application, and other EMF characteristics. We tried to fix this issue by comparing the results of studies on each disease, but we failed due to the high diversity and lack of studies with similar exposure characteristics. Therefore, future research must concentrate on determining the optimal EMF parameters, such as frequency, intensity, and duration of treatment. In addition, future studies should consider that different parameters activate different mechanisms of action; as a result, linking the optimal parameters with their respective mechanisms of action could enhance the efficacy of this new therapeutic option.

## Conclusion

 In conclusion, EMF-based therapies are currently attracting attention due to their safety and effectiveness. In this study, we reviewed all interventional studies evaluating the effects of EMFs on improving outcomes related to dental implantology. Most studies showed the beneficial effects of EMF exposure, which may encourage researchers and clinicians to further focus on non-invasive treatments for improving outcomes in dental implantology.

## Competing Interests

 The authors declare that they have no competing interests with regard to the authorship and/or publication of this article.

## Consent for Publication

 Not applicable.

## Data Availability Statement

 Not applicable.

## Ethical Approval

 Not applicable.

## Funding

 No funding was allocated to this work.
